# Association of dietary pattern and Tibetan featured foods with high-altitude polycythemia in Naqu, Tibet: A 1:2 individual-matched case-control study

**DOI:** 10.3389/fnut.2022.946259

**Published:** 2022-09-23

**Authors:** Jiaxue Cui, Duoji Zhaxi, Xianzhi Sun, Nan Teng, Ruiqi Wang, Yizhuo Diao, Chenxin Jin, Yongxing Chen, Xiaoguang Xu, Xiaofeng Li

**Affiliations:** ^1^Department of Epidemiology and Health Statistics, Dalian Medical University, Dalian, China; ^2^Institute of High Altitude Medicine, People’s Hospital of Naqu Affiliated to Dalian Medical University, Naqu, China; ^3^Department of Neurosurgery, Second Affiliated Hospital of Dalian Medical University, Dalian, China

**Keywords:** Tibet, dietary pattern, featured foods, high altitude polycythemia, 1:2 individual matched case-control study

## Abstract

This study focused on the association of dietary patterns and Tibetan featured foods with high-altitude polycythemia (HAPC) in Naqu, Tibet, to explore the risk factors of HAPC in Naqu, Tibet, to raise awareness of the disease among the population and provide evidence for the development of prevention and treatment interventions. A 1:2 individual-matched case-control study design was used to select residents of three villages in the Naqu region of Tibet as the study population. During the health examination and questionnaire survey conducted from December 2020 to December 2021, a sample of 1,171 cases was collected. And after inclusion and exclusion criteria and energy intake correction, 100 patients diagnosed with HAPC using the “Qinghai criteria” were identified as the case group, while 1,059 patients without HAPC or HAPC -related diseases were identified as the control group. Individuals were matched by a 1:2 propensity score matching according to gender, age, body mass index (BMI), length of residence, working altitude, smoking status, and alcohol status. Dietary patterns were determined by a principal component analysis, and the scores of study subjects for each dietary pattern were calculated. The effect of dietary pattern scores and mean daily intake (g/day) of foods in the Tibetan specialty diet on the prevalence of HAPC was analyzed using conditional logistic regression. After propensity score matching, we found three main dietary patterns among residents in Naqu through principal component analysis, which were a “high protein pattern,” “snack food pattern,” and “vegetarian food pattern.” All three dietary patterns showed a high linear association with HAPC (*p* < 0.05) and were risk factors for HAPC. In the analysis of the relationship between Tibetan featured foods and the prevalence of HAPC, the results of the multifactorial analysis following adjustment for other featured foods showed that there was a positive correlation between the average daily intake of tsampa and the presence of HAPC, which was a risk factor. Additionally, there was an inverse correlation between the average daily intake of ghee tea and the presence of HAPC, which was a protective factor.

## Introduction

High-altitude polycythemia (HAPC) is a clinical syndrome that occurs among natives or long-term residents living ≥ 2,500 m above sea level. It is characterized by excessive erythrocytosis (in women, hemoglobin > 19 g/dL; in men, hemoglobin > 21 g/dL), severe hypoxemia, and (in some cases) moderate or severe pulmonary hypertension, which may evolve to cor pulmonale, leading to congestive heart failure (1). The 2004 definition of the disease considers HAPC and chronic plateau disease (CMS) to be the same thing. However, in naming, typing, and diagnosing altitude sickness in China (2), HAPC is considered to be a separate clinical subtype, whereas CMS refers to the clinical manifestations of both HAPC and high-altitude heart disease (3). In our study, HAPC was considered to be a separate clinical subtype to study its associated risk factors.

Excessive erythrocytosis is accompanied by excessive blood volume and extensive vasodilatation. Due to excessive red blood cells and hypoxemia, patients present with profound cyanosis. Characteristic pestle-like fingers and toes can usually be observed, with deep cyanosis of the oral and throat mucosae (4–6). Undoubtedly, the clinical features described above impose a serious disease burden and economic burden on patients with HAPC.

In the Tibetan Plateau, HAPC occurs in 5–18% of the total population (5, 6). HAPC affects people in the plateau region, but not all people in the plateau region have a high probability of developing HAPC. Studies have shown that local high-altitude populations have a unique set of heritable traits that can allow them to tolerate hypoxia, though between 1.2 and 33% of high-altitude populations globally are not adapted to this low-oxygen environment. Other research have shown that the prevalence of high-altitude erythrocytosis is not only related to genetic factors but also to the altitude, length of residence, age, and gender of the population (7, 8). Naqu, Tibet, is located in northern Tibet, with an average altitude of > 4,500 m. Its residents live in a harsh environment of low-pressure oxygen deprivation, high altitude, low temperature, and high ultraviolet light exposure for a long time (9). Therefore, the diet of Tibetans is very characteristic (10). Their featured foods are mainly ghee, tsampa, sweet tea, ghee tea, milk dregs, Tibetan cheese, and barley wine.

Currently, there is no study on the association of dietary patterns and Tibetan featured foods with HAPC. In this study, answers to the Health Status and Health Needs of People in Highland Areas Questionnaire and the 70-item Food Frequency Questionnaire (FFQ), which were modified according to the characteristics of the Tibetan diet, were collected from patients attending a tertiary care hospital in Naqu, Tibet, from December 2020 to December 2021, from which case and control groups were selected for analysis. Our study focused on the relationship between dietary patterns and Tibetan featured foods with HAPC in Naqu, Tibet, to explore the risk factors of HAPC in Naqu, Tibet, to raise awareness of the disease among the population, provide evidence for the development of prevention and treatment interventions, and guide people to eat scientifically.

## Materials and methods

### Study subjects

Residents of three villages in the Naqu region of Tibet were selected as the study population. A face-to-face questionnaire was administered to the study population from December 2020 to December 2021. A sample of 1,171 cases was collected, including 100 cases in the case group and 1,071 cases in the control group.

We enrolled patients (1) with HAPC who were diagnosed by a tertiary care hospital in Naqu, Tibet, between December 2020 and December 2021; (2) who completed all anthropobiological measurements and questionnaires, including lifestyle characteristics as well as dietary assessments; (3) without missing data for the required variables; and (4) with hemoglobin > 19 g/dL (women) or hemoglobin > 21 g/dL (men) (1). Those with true erythrocytosis, other types of secondary erythrocytosis, or other HAPC complications (2) were excluded.

Enrollees in the control group met the same inclusion and exclusion criteria as the case group, with no abnormal blood test results and no true erythrocytosis, other secondary erythrocytosis, or other HAPC complications.

### Data collection

The data of cases and controls were collected through the Questionnaire on Health Status and Health Needs of People in Highland Areas and a reliability-validated FFQ (11) (Supplementary Data 1), which were modified according to the characteristics of the Tibetan diet, by the investigator in face-to-face interviews with patients. At least two investigators were Tibetan and could communicate in both the native Tibetan language and Mandarin. Subjects filled in the FFQ according to their average intake per year, month, week, or day, which was converted to the average daily intake for statistical purposes. A physical examination, including the measurement of height and weight, was performed, and we calculated BMI as weight (kg) divided by height (m) squared. The data-collection process ensured that all participants completed questionnaires, and the final factors included in the study were (Table 1) gender, age, BMI, smoking status, alcohol status, length of residence, working altitude, and average daily frequency of intake of each food item (g/day). We excluded foods with an intake of less than 0.5 g/d, finally leaving 70 kinds of food. In the meantime, we calculated the average daily total energy intake, protein intake, carbohydrate intake, and fat intake for each person according to the Chinese Food Composition Tables (12). And corrected the intake of each food according to the mean value of total energy by the residual method. After combining previous studies, we excluded data from samples with a daily energy intake of less than 450 kcal or more than 5,000 kcal (13–15). Therefore, 12 participants were excluded for reporting implausible energy intake. Finally, we determined 1,159 samples, including 100 cases in the case group and 1,059 cases in the control group. This study was approved by the Ethics Review Committee of Naqu People’s Hospital, and all participants signed a consent form before completing the interview.

**TABLE 1:** PSM of the name of each variable and the meaning of the assigned value.

Variable	Variable meaning	Variable assignment
Age	The age of the research subjects	
Gender	Gender of study subjects	1: Male, 2: Female
Body mass index	Subject’s height (m) divided by weight squared (kg)	
Smoke	Smoking status	1: Yes, 0: No
Alcohol	Alcohol status	1: Yes, 0: No
Length of residence	Years of residence in the Naqu region of Tibet	1: From birth, 2: Migrate
Working altitude	Usually performs work at the altitude	1: < 4,500 m, 2: > 4,500 m

### Propensity score matching

After referring to previous literature, we performed 1:2 PSM using R 4.1.2 to exclude the influence of confounding factors on dietary patterns and featured foods. The case and control groups were individually matched 1:2 according to gender, age, body mass index (BMI), length of residence, working altitude, smoking status, and alcohol consumption according to the propensity score matching (PSM) method to reduce confounding bias. The 1:2 match of case and control groups was completed, involving 1,059 controls, based on gender, age, BMI, length of residence, working altitude, smoking status, and alcohol status for 100 case groups. To ensure that the 100 cases in the case group were matched to 200 controls, the caliper distance was selected to be as small as possible. Propensity scores were calculated using a logistic regression model, and to try to match all cases and ensure a low caliper distance (16), the final matching was done at a caliper distance of 1.5. The standardized mean difference (SMD) was used to assess the equilibrium of baseline information between the case and control groups after PSM. A SMD of 0.1 usually indicates a good balance and can be considered a tiny difference between the case and control groups.

### Identification of dietary patterns

Based on the intake frequency of each food item in the dietary survey questionnaire, the average daily food intake (g/day) was calculated, energy correction based on total energy intake, and 70 food items were divided into 14 food groups according to the similarity of types and nutrient composition, including “staple foods,” “meat,” “milk and milk products,” “eggs,” “beans,” “dishes,” “snacks and nuts,” “fungi and mushrooms,” “all vegetables,” “fruits,” “tea and beverages,” “sugars,” “oils,” and “salt” (Table 2). Then, the dietary intake frequencies of each food group were calculated, and the data were standardized to facilitate principal component analysis for dimensionality reduction (16–18). Principal component analysis was done using R 4.1.2 and GraphPad Prism 9. The dietary patterns were selected according to the parallel analysis scree plot, and the factor loadings accounted for by the food groups were also calculated. Dietary patterns were defined according to the common characteristics of the top three factors, accounting for the factor loadings of each dietary pattern. The score for each dietary pattern was calculated for each study subject, with a high score representing greater adherence to the dietary pattern and a low score representing less adherence to the dietary pattern. On this basis, we analyzed the association between the prevalence of HAPC and dietary pattern scores in Naqu, Tibet. In addition, we selected six kinds of Tibetan food items, namely “tsampa,” “ghee,” “milk dregs,” “cheese,” “sweet tea,” and “ghee tea,” and analyzed the relationship between Tibetan featured foods and HAPC.

**TABLE 2:** FFQ groups and foods contained and the average intake after energy correction of the two groups (before PSM).

Group	Foods contained and average intake of the two groups*
Staple foods	Rice (41.85, 49.49), tsampa (64.83, 35.75), wheat flour (19.92, 8.40), rice flour (17.34, 19.78), ginseng fruit (0.03, 2.30), hanging noodles (2.21, 9.72), corn (2.92, 8.67), sweet potato (0.92, 0.47)
Meat	Pork (muscle) (–3.41, 11.06), pork (fat and muscle) (–5.19, 2.91), beef (–6.93, 67.12), lamb (31.26, 7.94), chicken (2.04, 11.48), sausage (–1.44 3.13), pork legs, and feet (0.01, 0.22)
Milk and milk products	Whole fresh milk (30.87, 22.83), low-fat fresh milk (5.95, 6.33), fresh goat’s milk (6.61, 2.16), yogurt (12.81, 10.39), ice cream (5.06, 4.65), cheese (4.69, 10.61), milk dregs (5.47, 5.19), ghee (–17.48, 19.81)
Eggs	Eggs (–11.37, 12.97), duck eggs (1.17, 0.42)
Beans	Tofu (5.74, 7.27), dry bean-curd (0.00, 2.29), soy milk (1.35, 2.29)
Savory dishes	Salted radish (4.29, 1.58), salty cucumber (4.35, 0.72), mustard (2.65, 0.55), pickle (6.76, 1.21)
Snacks and nuts	Cakes (–2.88, 4.01), bread (4.32, 7.68), cookies (3.05, 4.01), instant noodles (13.14, 5.52), potato chips (0.07, 1.35), peanuts (21.31, 4.17), walnuts (1.60, 1.09), chestnuts (–0.13, 0.57)
Fungi and mushrooms	Dried mushrooms (0.46, 4.08), fresh mushrooms (2.97, 1.37), kelp (4.86, 0.75), nori (7.29, 0.78)
All vegetables	Cabbage, carrot, potato, cabbage, green pepper, bean sprout, lettuce, cauliflower, bell pepper, winter squash, pumpkin (Due to the wide variety of vegetables, only the average daily intakes of the major vegetable categories were collected in the questionnaire design and survey, and these vegetables mentioned in the table were only for the respondents’ reference recall to obtain the most realistic data. Vegetables were also counted as a category only in the process of counting FFQ food types and quantities.) (115.01, 48.36)
Fruits	Watermelon (24.50, 11.00), grapes (5.16, 8.21), apples (59.43, 23.09), bananas (76.25, 9.85), pears (53.36, 5.95), oranges (33.13, 13.07), raisins (0.68, 2.08), peaches (2.36, 1.40), strawberries (–0.13, 1.43), lychees (26.59, 0.21), mangoes (0.03, 1.15), hawthorn (0.59, 4.40), persimmons (8.15, 0.22), dates (5.30, 0.45)
Tea and beverages	Ghee tea (40.01, 99.73), sweet tea (12.50, 13.11), green tea (62.90, 69.94), cola (34.33, 22.93), juice (26.69, 9.04), coffee (2.22, 1.97), black tea (–1.16, 3.00), milk tea (3.32, 3.91)
Sugars	Sugar (26.44, 6.24) (The sugar in this case is refined sugar made from molasses extracted from sucrose and beets.)
Oils	Peanut oil (15.25, 16.09)
Salt	Edible salt (7.47, 10.17)

*The numbers in parentheses represent the average intake of each food in the case and control groups, respectively, after energy correction. Intake units in g/day. Example: food (mean intake for the case group, mean intake for the control group).

### Statistical analysis

Data were entered using Epidata 3.1, and all data were collated and analyzed using R 4.1.2. Energy-corrected food group intakes were computed using the residual method. A *t*-test was used for continuous variables, and the chi-squared test was used for categorical variables to describe the differences between groups. Univariate and multivariate conditional logistic regression analyses were performed to analyze the association of dietary pattern scores and Tibetan specialty foods with HAPC. We calculated the odds ratio (OR) with 95% confidence interval (CI) values, and *p* < 0.05 was considered to be statistically significant (19, 20).

## Results

### Baseline characteristics and propensity score matching

A total of 1,171 Tibetan herdsmen were collected before matching in this study, and 1,159 cases were retained. About 12 participants were excluded for reporting implausible energy intake of less than 450 kcal or more than 5,000 kcal. The average total energy intake of the study subjects was 1,689.00 ± 839.25 kcal/d, the average daily intake of protein was 48.11 ± 34.16 g/d, the average daily intake of carbohydrates was 203.94 ± 120.32 g/d, and the average daily intake of fat was 76.14 ± 37.30 g/d. Therefore, we included 100 cases in the case group and 1,059 cases in the control group. And all cases were matched according to the PSM method to achieve 1:2 matching based on gender, age, BMI, length of residence, working altitude, smoking status, and alcohol status.

The baseline characteristics of the study subjects before and after matching are shown in Tables 3, 4. A *t*-test was used for continuous variables, and the chi-squared test was used for categorical variables to describe the differences between groups. The calculated *p*-values for all factors after matching were > 0.05 and the SMD values were < 0.1, which were considered indicative of good equilibrium between the case and control groups after matching.

**TABLE 3:** Baseline characteristics of study subjects before PSM.

		Control (*n* = 1,059)	Case (*n* = 100)	*p*	SMD
Age (years), mean (*SD*)		32.17 (13.50)	43.01 (17.43)	<0.001	0.695
BMI (kg/m^2^), mean (*SD*)		24.05 (5.77)	26.80 (5.04)	<0.001	0.508
Gender (%)	Male	603 (56.9)	40 (40.0)	0.002	0.344
	Female	456 (43.1)	60 (60.0)		
Length of residence (%)	From birth	834 (78.8)	99 (99.0)	<0.001	0.680
	Immigrated	225 (21.2)	1 (1.0)		
Working altitude (%)	< 4,500 m	360 (34.0)	6 (6.0)	<0.001	0.747
	> 4,500 m	699 (66.0)	94 (94.0)		
Smoking (%)	Yes	144 (13.6)	5 (5.0)	0.021	0.299
	No	915 (86.4)	95 (95.0)		
Alcohol (%)	Yes	129 (12.2)	10 (10.0)	0.631	0.070
	No	930 (87.8)	90 (90.0)		

BMI, body mass index, SD, standard deviation; SMD, standardized mean difference.

**TABLE 4:** Baseline characteristics of study subjects after PSM.

		Control (*n* = 200)	Case (*n* = 100)	*p*	SMD
Age (years), mean (*SD*)		41.65 (16.09)	43.01 (17.43)	0.501	0.081
BMI (kg/m^2^), mean (*SD*)		26.49 (6.51)	26.80 (5.04)	0.672	0.054
Gender (%)	Male	81 (40.5)	40 (40.0)	1.000	0.010
	Female	119 (59.5)	60 (60.0)		
Length of residence (%)	From birth	197 (98.5)	99 (99.0)	1.000	0.045
	Immigrated	3 (1.5)	1 (1.0)		
Working altitude (%)	< 4,500 m	12 (6.0)	6 (6.0)	1.000	<0.001
	> 4,500 m	188 (94.0)	94 (94.0)		
Smoking (%)	Yes	10 (5.0)	5 (5.0)	1.000	<0.001
	No	190 (95.0)	95 (95.0)		
Alcohol (%)	Yes	21 (10.5)	10 (10.0)	1.000	0.016
	No	179 (89.5)	90 (90.0)		

BMI, body mass index; SD, standard deviation; SMD, standardized mean difference.

In the case group, the average age was 43.01 ± 17.43 years, the average BMI was 26.80 ± 5.04 kg/m^2^, there were 40 (40.0%) men and 60 (60.0%) women, and 99 (99.0%) participants had lived in the Naqu region of Tibet since birth. The number of people who usually worked at an altitude of > 4,500 m was 94 (94.0%). The number of smokers was 5 (5.0%) and the number of drinkers was 10 (10.0%).

In the control group, the mean age before matching was 32.17 ± 13.50 years, the mean BMI was 24.05 ± 5.77 kg/m^2^, there were 603 (56.9%) men and 456 (43.1%) women, and 834 (78.8%) participants had lived in the Naqu area of Tibet since birth. The number of people who usually worked at an altitude of > 4,500 m was 699 (66.0%). The number of smokers was 144 (13.6%) and the number of alcohol drinkers was 129 (12.2%).

After matching, the mean age of the control group was 41.65 ± 16.09 years, the mean BMI was 26.49 ± 6.51 kg/m^2^, there were 81 (40.5%) men and 119 (59.5%) women, and 197 (98.5%) participants had lived in the Naqu area of Tibet since birth and 3 (1.5%) had migrated here. The number of people who usually worked at an altitude of > 4,500 m was 188 (94.0%). The number of smokers was 10 (5.0%) and the number of drinkers was 21 (10.5%).

### Identification of dietary patterns

**FIGURE 1:**
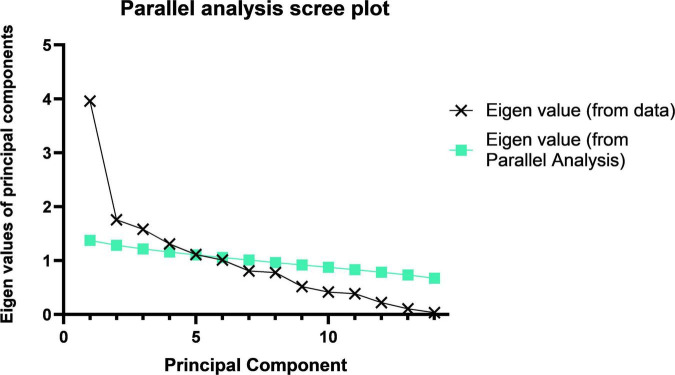
Parallel analysis scree plot. The principal component analysis of the 14 food groups and the component folds of the actual data and their slopes indicate that we should choose three principal components.

According to the recommendations of the parallel analysis scree plot (Figure 1), 14 food groups were grouped into three dietary patterns. According to the scores derived from the factor loadings (Table 5), the top three food groups in the factor loadings of the first dietary pattern were eggs (0.93), meats (0.92), and milk and milk products (0.90), which were significantly characterized as being rich in protein, thus defining such dietary patterns as a “high protein pattern.” The top three factor loadings of the second dietary pattern were snacks and nuts (0.81), fruits (0.76), and beans (0.64), which are not the main source of energy intake in the general sense (not staple foods, meat, or vegetables), so we defined them in general as a “snack food pattern.” The top three factor loadings of the third dietary pattern were fungi and mushrooms (0.80), tea and beverages (0.58), and all vegetables (0.45), which were characterized by the absence of animal-based foods and belong to the vegetarian mode and therefore indicative of a “vegetarian food pattern.” The variance contributions of these three dietary patterns were 0.26, 0.14, and 0.12, respectively, and their cumulative variance contribution was 0.52.

**TABLE 5:** Dietary pattern factor loadings.

Factor	High protein pattern	Factor	Snack food pattern	Factor	Vegetarian food pattern
Eggs	0.93	Snacks and nuts	0.81	Fungi and mushrooms	0.80
Meat	0.92	Fruits	0.76	Tea and beverages	0.58
Milk and milk products	0.90	Beans	0.64	All vegetables	0.45
Savory dishes	0.80	Savory dishes	0.12	Savory dishes	0.34
Staple foods	0.52	Fungi and mushrooms	0.09	Sugars	0.21
Salt	0.23	All vegetables	0.06	Fruits	0.14
All vegetables	0.01	Sugars	0.05	Beans	0.03
Fungi and mushrooms	0.00	Salt	0.01	Milk and milk products	0.00
Snacks and nuts	–0.07	Eggs	–0.15	Snacks and nuts	–0.12
Tea and beverages	–0.08	Milk and milk products	–0.16	Eggs	–0.14
Beans	–0.16	Meat	–0.23	Meat	–0.14
Fruits	–0.17	Staple foods	–0.23	Salt	–0.22
Sugars	–0.20	Oils	–0.26	Oils	–0.25
Oils	–0.25	Tea and beverages	–0.36	Staple foods	–0.32

### Association between dietary patterns and high-altitude polycythemia

The results from before and after adjusting for the scores of the other two dietary patterns showed (Table 6) that greater adherence to the snack food pattern and vegetarian food pattern had a significant linear correlation with the prevalence of HAPC (*p* < 0.001). In the analysis of the high protein pattern, the univariate analysis did not show a statistically significant difference, but after adjusting for the other two dietary patterns, the high protein pattern showed an association with the prevalence of HAPC. Univariate conditional logistic regression analysis showed that, for each 1-unit increase in adherence to the snack food pattern (OR, 1.84; 95% CI, 1.34–2.53), the risk of developing HAPC increased by 1.84 times; whereas the vegetarian food pattern (OR, 2.47; 95% CI, 1.73–3.54) increased the risk of HAPC by 2.47 times for each additional 1 unit of compliance.

**TABLE 6:** Conditional logistic regression analysis of dietary pattern scores.

	Control (*n* = 200)	Case (*n* = 100)	Crude		Adjusted^†^	
				
	Mean (*SD*)	Mean (*SD*)	OR (95% CI)	*p*	OR (95% CI)	*p*
High protein pattern	–0.05 (1.05)	0.11 (0.89)	1.18 (0.92–1.51)	0.202	1.52 (1.13–2.04)	0.005**
Snack food pattern	–0.18 (1.13)	0.35 (0.51)	1.84 (1.34–2.53)	<0.001***	2.11 (1.51–2.94)	<0.001***
Vegetarian food pattern	–0.24 (1.06)	0.48 (0.64)	2.47 (1.73–3.54)	<0.001***	2.80 (1.94–4.06)	<0.001***

CI, confidence interval; SD, standard deviation; OR, odds ratio. ^†^Multifactorial conditional logistic regression, adjusted for scores of the other two dietary patterns, *p < 0.05, **P < 0.1, ***p < 0.001.

The multifactorial conditional logistic regression analysis showed that, after adjusting for the other two dietary patterns, the OR of the high protein pattern score was 1.52 (1.13–2.04), the OR of the snack food pattern score was 2.11 (1.51–2.94), and the OR of the vegetarian food pattern score was 2.80 (1.94–4.06). All three dietary patterns were risk factors affecting the prevalence of HAPC.

### Association between Tibetan featured foods and high-altitude polycythemia

The results of the univariate conditional logistic regression analysis showed (Table 7) that tsampa (OR, 1.05; 95% CI, 1.03–1.06) increased the risk of HAPC by 1.05 times for each 1-unit increase in daily intake. In addition, cheese (OR, 0.95; 95% CI, 0.93–0.98) was associated with a 0.95-fold reduction in the risk of HAPC for each additional 1 unit of daily intake, and ghee tea (OR, 0.99; 95% CI, 0.98–0.99) was associated with a 0.99-fold reduction in the risk of HAPC for each additional 1 unit of daily intake. Ghee (OR, 1.00; 95% CI, 0.99–1.00) had no effect on this disease. No correlation was found between milk dregs or sweet tea and the prevalence of HAPC.

**TABLE 7:** Conditional logistic regression analysis of Tibetan featured foods.

	Control (*n* = 200)	Case (*n* = 100)	Crude		Adjust^†^	
				
	Mean (*SD*)	Mean (*SD*)	OR (95% CI)	*p*	OR (95%)	*p*
Tsampa	34.80 (26.35)	64.83 (22.09)	1.05(1.03–1.06)	<0.001***	1.04 (1.03–1.05)	<0.001***
Cheese	11.08 (15.07)	4.69 (5.55)	0.95 (0.93–0.98)	<0.001***	0.99 (0.95–1.03)	0.495
Milk dregs	5.07 (13.05)	5.47 (4.07)	1.00 (0.98–1.03)	0.768	1.01 (0.97–1.04)	0.776
Ghee	18.26 (107.84)	–17.48 (89.46)	1.00 (0.99–1.00)	0.009**	1.00 (0.99–1.00)	0.292
Ghee tea	98.93 (65.83)	40.01 (55.65)	0.99 (0.98–0.99)	<0.001***	0.99 (0.98–1.00)	0.002**
Tibetan sweet tea	12.63 (28.83)	12.50 (15.69)	1.00 (0.99–1.01)	0.962	1.00 (0.99–1.01)	0.947

CI, confidence interval; SD, standard deviation; OR, odds ratio. ^†^Multifactorial conditional logistic regression, adjusted for mean daily intake of other featured foods, *p < 0.05, **p < 0.1, ***p < 0.001.

In the multifactorial conditional logistic regression analysis, after adjusting for the average daily intake of several other specialty foods, tsampa (OR, 1.04; 95% CI, 1.03–1.05) was associated with a 1.04-fold increased risk of HAPC for each 1-unit increase in daily intake, while ghee tea (OR, 0.99; 95% CI, 0.98–1.00) was associated with a 0.99-fold reduction in the risk of HAPC for each 1-unit increase in daily intake.

The combined results of the univariate and multifactorial analyses concluded that tsampa intake is a risk factor for the development of HAPC, while ghee tea intake is a protective factor for HAPC.

## Discussion

Our study focuses on the association of dietary patterns and Tibetan featured foods with HAPC in Naqu, Tibet, to explore the risk factors of HAPC in Naqu, Tibet, to raise awareness of the disease among the population and provide evidence for the development of prevention and treatment interventions. In this study, the 70 food items in the food frequency questionnaire were grouped into 14 food groups according to the similarity of food types and nutrient contents, and downscaled into three dietary patterns—namely, a “high protein pattern,” “snack food pattern,” and “vegetarian food pattern.” According to the principal components analysis of each dietary pattern of the study subjects, univariate and multivariate conditional logistic regression analyses were conducted, respectively. Then, the average daily intakes of six foods with Tibetan characteristics (tsampa, ghee, cheese, milk dregs, sweet tea, and ghee tea) were analyzed to determine their effect on HAPC among Tibetan residents in the Naqu area.

### Selection of matching variables in propensity score matching

Previous research have indicated that high-altitude adaptation occurs at the expense of being more prone to CMS (21). Lorenzo et al. (22) demonstrated that the *EGLN1* haplotype in Tibetans is associated with protection from polycythemia (22). Simonson et al. (7) suggested that Tibetan high-altitude adaptation is not determined by a single gene but instead by multiple evolved genetic adaptations acting in concert with each other. Three of these genes, *EPAS1*, *EGLN1*, and *PPARA*, regulate or are regulated by the hypoxia-inducible factor, a principal controller of erythropoiesis and other organismal functions (7). Hurtado et al. (23) demonstrated that variants in *EPAS1* correlate with lower hemoglobin concentrations, supporting their roles in maintaining a blunted erythropoietic response to lower oxygen saturation values, which is a hallmark of altitude adaptation in Tibetans (23). Julian et al. (24) demonstrated that perinatal hypoxia increases susceptibility to HAPC (24). These demonstrations of adaptation to high altitude suggested to us that genetics plays an important role in the development of HAPC among Tibetans due to their adaptation to altitude, and those individuals who newly migrate to the area are more likely to develop HAPC. Zhang et al. (25) demonstrated that an elevation of around 4,500 m represented a turning point for Tibetans, with a dramatic increase in both hemoglobin concentration and polycythemia prevalence (25). In the Naqu region of Tibet, the average altitude is > 4,500 m, and our grouping for working altitude was also bounded by 4,500 m to eliminate the confounding bias brought by altitude. In addition, the influencing factors of HAPC recognized by the medical community are gender, age, smoking status, and alcohol consumption: men are more likely to have the disease than women, older people are more likely to have the disease, and smoking and alcohol consumption could also lead to the development of HAPC.

In summary, we selected confounding factors, such as gender, age, BMI, length of residence, work altitude, smoking status, and alcohol status, which may have influenced the prevalence of HAPC in previous studies, as PSM variables to achieve a 1:2 match for all cases.

### Association between dietary patterns and high-altitude polycythemia

The results of the principal component analysis showed that the 14 food groups were suggested to be interpreted in three patterns which were termed a “high protein pattern,” “snack food pattern,” and “vegetarian food pattern,” respectively, according to their top three highest factor loadings. In previous studies, the Tibetan diet has been characterized by high meat and low vegetable intake, and Tibetan residents follow a high grain and meat intake and high fat and sodium diet pattern (10). The contribution of variance of the “high protein pattern” was 0.26, which was the highest among the three dietary patterns—that is, it was the most important among the three dietary patterns. Among them, the factor loadings of eggs, meat, and milk and milk products were all as high as 0.9 or more. This dietary pattern was not only the most dominant but also has distinctive features and was very characteristic of Tibet. In the snack food pattern, snack foods, fruits, and beans all had factor loadings greater than 0.6, and they were not universally satiating foods. They were eaten mostly between meals or with meals. In the last “vegetarian food pattern,” the top three factor loadings were fungi and mushrooms, tea and beverages, and vegetables. This is a dietary pattern without animal food, which may be related to religious beliefs.

**FIGURE 2:**
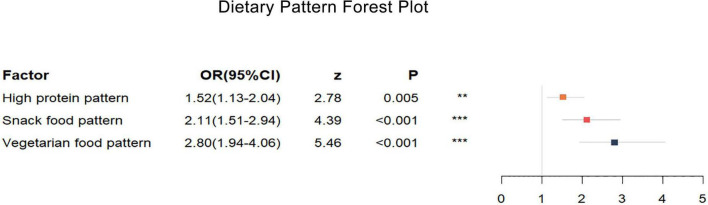
Dietary pattern forest plot. High protein pattern, snacking pattern, and vegetarian pattern all increase the risk of high-altitude polycythemia. The different colored rectangles represent different dietary patterns. The horizontal coordinate represents the OR value, and the length of the thin line indicates the size of the 95% CI. Multifactorial conditional logistic regression, adjusted for scores of the other two dietary patterns, *P < 0.05, **P < 0.1, ***P < 0.001.

**FIGURE 3:**
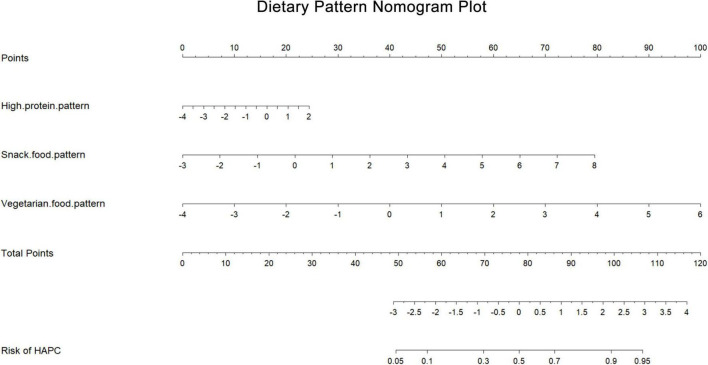
Dietary pattern nomogram plot. Based on the contribution of each dietary pattern to the prevalence of HAPC in the conditional logistic regression analysis, a score was assigned to each intake level of each dietary pattern. The individual scores were then summed to obtain the total score, which resulted in the predicted value of different dietary pattern intake levels in relation to the prevalence of HAPC.

Our study showed that the mean protein and carbohydrate intakes of Tibetans were lower than the recommended intakes (26), while the total energy intake of Tibetans was also low. This is quite different from the results of a 1992 study of Naqu pastoralists (27). However, we also found that in the 1992 study, the intake of vegetables among the Naqu pastoralists was only 1.8 g/d. In our study, the intake of vegetables was 115.01 g/d and 48.36 g/d. And the intake of staple foods such as tsampa, flour, and rice all differed significantly from the 1992 findings. The intake of vegetables by the population of Nagqu has greatly increased and the intake of staple foods has decreased, and the change in dietary structure may be the main reason for the decrease in total energy intake in our study. However, there has been a decreasing trend in energy intake among residents in Tibet in recent years (28). Our results of lower energy intake and protein intake are similar to some previous studies (17, 29). However, their fat intake was higher than the recommended intake and the findings in 1992. This may be related to a variety of factors such as the vast and sparsely populated Tibet, lack of resources, religious beliefs, long, cold winters, and a low-oxygen environment. In addition to that, Tibetans may reduce the intake and consumption of energy and protein, and increase the intake of fat to keep warm at the same time in recent year. And from these three dietary patterns and energy intake, it was easy to see that the residents in this area still follow the traditional high-protein and high-fat dietary pattern of the Tibetan region. With both total energy intakes below the recommended values, Tibetans still followed a dietary pattern based on eggs, meats, and milk. The dietary variety of residents has become richer by analyzing dietary patterns, which is different from the previously focused intake of Tibetan specialty foods (17). In our results, all three dietary patterns were risk factors for the prevalence of HAPC (Table 6). A forest plot (Figure 2) shows the ORs and 95% CIs for the dietary pattern scores for the prevalence of HAPC, and a nomogram plot (Figure 3) shows the hazard predictions for the prevalence of HAPC for the three dietary model scores.

The cumulative variance contribution of these three dietary patterns reached 0.52, explaining the dietary patterns of more than half of the population, but we believed that the dietary characteristics represented by these three dietary patterns have certain limitations and do not facilitate a balanced diet, that is, the currently advocated for the Mediterranean dietary pattern (30, 31). With the development of the Tibetan economy and infrastructure, the dietary characteristics of the Tibetan region have gradually changed, and residents’ dietary patterns now tend to be more balanced, but an unhealthy dietary pattern is still a risk factor for HAPC. In addition, only the frequency of food intake was counted in our questionnaire; the analysis did not incorporate the specific processing of these foods by residents, and it was shown in both our study and previous ones that people in Tibetan areas have the cooking characteristics of grilling and high levels of salt addition (32). This may also affect the relationship between dietary patterns and the prevalence of HAPC. In addition, Lotti et al. demonstrated that the morning chronotype is associated with higher adherence to the Mediterranean diet (33), while Gokhale and Rao suggested that income affects dietary diversity and dietary patterns (34). These studies offer us some hints that both diet timing and income may impact dietary patterns, which may contribute to the risk of dietary patterns causing HAPC.

### Association between Tibetan featured foods and high-altitude polycythemia

The Tibetan region of China has a special alpine climate and lack of oxygen, and to adapt to this climate, Tibetans have a unique lifestyle and unique regional dietary characteristics (35). The unique regional diet of Tibetans is strongly influenced by the biogeography of the region, indigenous traditions, popular religious beliefs, and dietary taboos (17). Studies had shown that about 25% of the foods regularly consumed by Tibetans are traditional Tibetan foods, such as tsampa, yak meat, and ghee tea (11). In our study, six foods with Tibetan characteristics, namely tsampa, ghee, sweet tea, ghee tea, milk dregs, and cheese, were analyzed separately to correlate each with the prevalence of HAPC.

**FIGURE 4:**
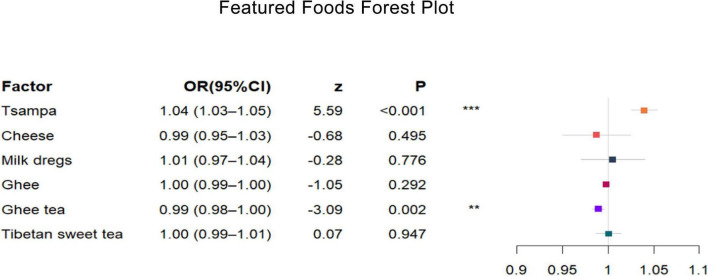
Featured foods forest plot. After adjusting several other featured foods, tsampa intake increases the risk of high-altitude polycythemia, while ghee tea intake decreases the risk of high-altitude polycythemia. The different colored rectangles represent different featured foods. The horizontal coordinate represents the OR value, and the length of the thin line indicates the size of the 95% CI. Multifactorial conditional logistic regression, adjusted for mean daily intake of other featured foods, *P < 0.05, **P < 0.1, ***P < 0.001.

**FIGURE 5:**
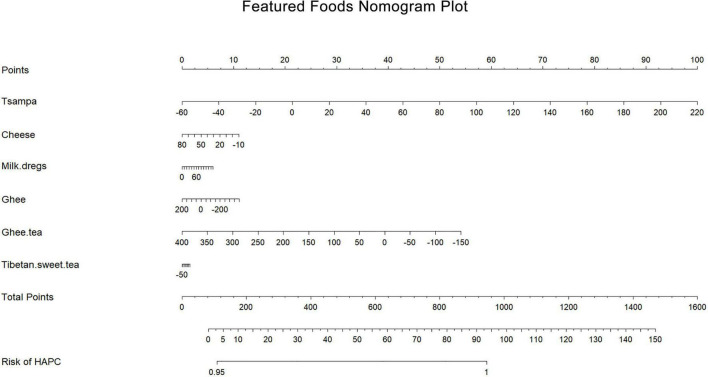
Featured foods nomogram plot. Based on the contribution of each featured food to the prevalence of HAPC in the conditional logistic regression analysis, a score was assigned to each intake level of each featured food. The individual scores were then summed to obtain the total score, which resulted in the predicted value of different featured food intake levels in relation to the prevalence of HAPC.

The results of a univariate logistic regression analysis of Tibetan specialty foods and HAPC showed that tsampa, cheese, ghee, and ghee tea were associated with the prevalence of HAPC, and cheese and ghee tea had protective effects. After adjusting for other featured foods, multifactorial logistic regression analysis showed that tsampa and ghee tea still affected the prevalence of HAPC: tsampa (OR, 1.04; 95% CI, 1.03–1.05) was a risk factor and ghee tea (OR, 0.99; 95% CI, 0.98–1.00) was a protective factor. A forest plot (Figure 4) shows the ORs and 95% CIs for the mean daily intake of the Tibetan featured foods according to the prevalence of HAPC, and a nomogram plot (Figure 5) shows the predicted risk of the mean daily intake of the featured foods relative to the prevalence of HAPC.

These Tibetan-featured foods each have their own special nutritional value. The nutrients in tsampa are mainly crude protein, crude fat, crude cellulose, total sugar, amino acids, and minerals (36). Ghee is also a typical yak dairy product, similar to butter, that is extracted from yak milk, and Tibetans consume yak and its dairy products to supplement their protein and energy intake. Compared to common butter, ghee has greater functional and nutritional values, mainly because of its higher unsaturated fatty acid content and richer functional lipids than common butter (37, 38). There are many cows and sheep in Tibetan pastoral areas, and there are also many dairy products. Among them, the representative ones are milk dregs and cheese. Milk dregs are substances left over after milk is refined from ghee; after boiling and water evaporation, what remains are milk dregs (39). The types of cheese consumed by Tibetan residents are mostly yak milk cheese, which is rich in nutritional value due to its special geographical location and climatic conditions and contains large amounts of protein, amino acids, lactose, and minerals (40). Ghee tea and sweet tea both have the function of tea in Tibet. Sweet tea is made by adding fresh milk or milk powder, white enamel, and a little salt to black tea. Ghee tea is made with ghee and brick tea and a certain amount of salt. Ghee tea and sweet tea can keep the drinker warm, replenish their energy, fight plateau reactions, and mitigate the lack of vitamins due to the low intake of fruits and vegetables (41). Tea is rich in tea polyphenols and catechins, and it is generally believed that these nutrients have a positive effect on the body by reducing blood lipids and blood pressure. Studies have shown that ghee has a certain effect on blood lipids. However, the effect of ghee tea on blood lipids is not obvious, and some studies have also shown that ghee can increase the concentration of bound catechins by decreasing the concentration of free catechins in plasma, thus altering the metabolism of catechins in the body (42, 43). These studies suggest that the effects of ghee and ghee tea on the prevalence of some diseases are not uniform among Tibetan residents in highland areas. Therefore, in this study, ghee and ghee tea were analyzed separately.

When combining our findings with previous literature, it is clear that these foods have a special protective role against many diseases. This point leads us to speculate that these Tibetan-featured foods have special nutrients that are protective against many diseases. The statistical results showed that the effect of these foods on the prevalence of HAPC was not significant, with OR values close to 1. We believe the reason for this is that the prevalence of HAPC is not influenced by the intake of featured foods alone. The intake of featured foods is common throughout the Tibetan region, but the dietary pattern is not the same for each family or individual. To explore the relationship between dietary factors and HAPC, we should not only consider it in terms of specialty foods but also return to the macroscopic dietary pattern.

In this study, we did not investigate factors such as food preparation methods and daily meal duration. The effects of food preparation methods, daily meal duration, and income on dietary patterns and thus the causes of HAPC need to be investigated. Based on the results of this study, to reduce the prevalence of HAPC, we advocate that Tibetan residents should increase their dietary diversity and balance, not eat only vegetables and fruits, eat fewer snacks, and have three regular meals; process food as simple as possible and use local, seasonal fresh fruits and vegetables as ingredients to avoid loss of trace elements and antioxidant components; use vegetable oils (containing unsaturated fatty acids) instead of animal oils (containing saturated fatty acids) in cooking.

In recent years, the economic level of the Tibetan region has developed rapidly, living conditions have improved, and food variety in the diet has increased, but many living in the Tibetan region still prefer featured foods such as ghee, ghee tea, tsampa, milk dregs, and sweet tea, and the traditional dietary habits of high protein and high-fat intakes have not changed significantly. Our study is the first to focus on the effects of dietary patterns and featured foods on the prevalence of HAPC, and the results of our analysis also show that Tibetan dietary patterns and specialty diets are independent risk factors for HAPC in the Tibetan region.

### Strengths and limitations

Our study is the first to focus on the association of dietary patterns and Tibetan-featured foods with HAPC in the Naqu region of Tibet. We included Tibetan featured foods in the FFQ to make it more regional, and the conduct of a 1:2 individual-matched case-control study makes the analysis results more convincing.

The limitations in this study are as follows: in the analysis of dietary patterns, because of the structure of the questionnaire and the excessive variety of foods, each food could only be included in large food groups and then identified in the dietary models, making the analysis not detailed enough to accurately locate the foods that play a major influence on each dietary pattern. And we may not have included many foods in this study, thus resulting in low energy intake levels. Additionally, the cooking methods of foods and the daily meal duration of the population were not investigated. Finally, alcohol intake is an important factor affecting energy intake, and our FFQ did not address the investigation and analysis of alcohol intake. We have used PSM in our study to individually match for alcohol consumption, eliminating the effect of whether to drink alcohol on the prevalence of HAPC, and we were unable to determine the effect of alcohol intake on prevalence among drinkers.

## Conclusion

Unbalanced dietary intake of the high protein pattern, snacking pattern, and vegetarian pattern all increase the risk of HAPC. Tsampa intake increases the risk of HAPC, while ghee tea intake decreases the risk of HAPC.

## Data availability statement

The raw data supporting the conclusions of this article will be made available by the authors, without undue reservation.

## Ethics statement

The studies involving human participants were reviewed and approved by the Naqu People’s Hospital. The patients/participants provided their written informed consent to participate in this study. Written informed consent to participate in this study was provided by the participants’ legal guardian/next of kin.

## Author contributions

JC: original draft preparation, methodology, software analysis, formal analysis, and data curation. XL and XX: investigation, resources, manuscript review, editing, and supervision. All authors contributed to the article and approved the submitted version.
