# Supplementary material for: Association of dietary pattern and Tibetan featured foods with high-altitude polycythemia in Naqu, Tibet: A 1:2 individual-matched case-control study
Source: Front Nutr. 2022 Sep 23;9:946259. doi: 10.3389/fnut.2022.946259 (PMC9538783; doi:10.3389/fnut.2022.946259)
Supplement: Supplementary file 1 [file Data_Sheet_1.pdf]

Supplementary Material

1 Supplementary Data

Supplementary Data 1. The FFQ used during the investigation of this study

ཚོད་ལྟ་བྱེད་མཁན་གྱི་ཨང་གྲངས།
受试者编号：__________

རུས་མིང་།
姓名：__________

ཟས་རིགས་ཟ་ཚོད་བརྟག་དཔྱད་རེའུ་མིག
食物频率调查表

ཁྱེད་ནས་འདས་པའི་ལོ་གཅིག་གི་ནང་དུ་གཤམ་གྱི་ཟས་རིགས་ཆ་སྙོམས་ཟས་ཚོད་དང་གྲངས་ཀ་ལ་ཚོད་དཔག་གནང་དང་།
请填写您在过去一年中对下列食物的平均进食频率和数量：

E1ཟས་གཙོ་བོ། 主食	ཆ་སྙོམས་ཟས་ཚད་/ཁ 平均摄入量/克	ཆ་སྙོམས་ཟས་ཚད 平均摄入量/ཐེངས 次				
		ཐེངས་ 次/ཉིན 天	ཐེངས་ 次 /གཟའ་འཁོར 周	ཐེངས་ 次/ཟླ་བ 月	ཐེངས་ 次/ལོ། 年	གཏན་ནས་མེད 从不
		གནས་ཚུལ་དངོས་ལ་གཞིགས་ནས་འབྲི་དགོས། 按实际情况填写				填 99
1.01 འབྲས 大米						
1.02 རྩམ་པ 糌粑						
1.03གྲོ་ཕྱེ 小麦面粉						
1.04 འབྲས་ཕྱེ 米粉						
1.05 གྲོ་མ 人参果						
1.06 རྒྱ་ཐུག 挂面						
1.07 ཨ་ཤོམ 玉米						
1.08 ཁྲེ་རྒོད 高粱						
1.09ཞོག་ཁོག་དམར་པོ 红薯						
1.10ཆང་འབྲས 糯米						
1.11འབྲས 小米						
1.12འབྲུ་རིགས་གཞན 其他谷物 （**请注明：**________）						

E2ཤ་རིགས་དང་དེའི་བཟོས་རྫས 肉类及其制品	ཆ་སྙོམས་ཟས་ཚད་/ཁ 平均摄入量/克	ཆ་སྙོམས་ཟས་ཚད 平均摄入量/ཐེངས་ 次				
		ཐེངས་ 次/ཉིན 天	ཐེངས་ 次 /གཟའ་འཁོར 周	ཐེངས་ 次/ཟླ་བ 月	ཐེངས་ 次/ལོ 年	གཏན་ནས་མེད 从不

		གནས་ཚུལ་ལ་གཞིགས་ནས་འབྲི་དགོས། 按实际情况填写				填 99
2.01ཕག་ཤ 猪肉(ཤ་ཙན 肌肉)						
2.02ཕག་ཤ 猪肉（ཚིལ་ལུ་དང་ཤ་ཙན་ 脂肪和肌肉）						
2.03ཚགས་ཤ 牛肉						
2.04ལུག་ཤ 羊肉						
2.05བྱ་ཤ 鸡肉						
2.06ཤ་རྒྱངས་རྒྱུ་མ 香肠						
2.07 ཕག་ཤ 猪排						
2.08 ཕག་པའི་སུག་པ 猪腿和脚						
2.09 ཕག་པ་དང་དེ་མིན་སྲོག་ཆགས་གཞན་གྱི་མཆིན་པ 猪和其他动物肝脏						
2.10 ངང་པ་དང་ཁྱིམ་བྱ་གཞན 鸭，其他家禽						
2.11 ཚ་ཤ 咸肉						
2.12 དེ་མིན་དབང་པོ་གཞན 其他器官 **（请注明：________）**						

E3 འོ་མ་དང་འོ་ཟས་གཞན 牛奶和奶制品	ཆ་སྙོམས་ཟས་ཚད/ཁ 平均摄入量/克	ཆ་སྙོམས་ཟས་ཚད 平均摄入量/ཐེངས་ 次				
		ཐེངས་ 次/ཉིན 天	ཐེངས་ 次/གཟའ་འཁོར 周	ཐེངས་ 次/ཟླ་བ 月	ཐེངས་ 次/ལོ 年	གཏན་ནས་མེད 从不
		གནས་ཚུལ་དངོས་ལ་གཞིགས་ནས་འབྲི་དགོས། 按实际情况填写				填 99
3.01 འོ་མ་སོས་པ 全鲜奶						
3.02 ཚིལ་ཉུང་འོ་གསར 低脂鲜奶						
3.03 ལུག་གི་འོ་མ་སོས་པ 鲜羊奶						
3.04 ཞོ 酸奶						

3.05འཁྱགས་ཞོ 冰淇淋						
3.06ཪོ་ཕྱུར 奶酪						
3.07ཕྱུར་བ 奶渣						
3.08མར 酥油						
3.09དེ་མིན 其他 （**请注明：**________）						

E4སྒོ་ངའི་རིགས 蛋类	ཆ་སྙོམས་ཟས་ཚད/ཁེ 平均摄入量/克	ཆ་སྙོམས་ཟས་ཚད 平均摄入量/ཐེངས་ 次				
		ཐེངས 次/ཉིན 天	ཐེངས 次/གཟའ་འཁོར 周	ཐེངས་ 次/ཟླ་བ 月	ཐེངས་ 次/ལོ 年	གཏན་ནས་མེད 从不
		གནས་ཚུལ་དངོས་ལ་གཞིགས་ནས་འབྲི་དགོས། 按实际情况填写				填 99
4.01སྒོང་ང་ 鸡蛋						
4.02ངང་སྒོང་ 鸭蛋						
4.03 སྲེག་པའི་སྒོ་ང 鹌鹑蛋						
4.04སྒོ་ང་ནག་པོ 皮蛋						
4.05དེ་མིན 其他 （**请注明：**________）						

E5 སྲན་རིགས་ཐོན་རྫས 豆类产品	ཆ་སྙོམས་ཟས་ཚད/ཁེ 平均摄入量/克	ཆ་སྙོམས་ཟས་ཚད 平均摄入量/ཐེངས་ 次				
		ཐེངས་ 次/ཉིན 天	ཐེངས་ /གཟའ་འཁོར 周	ཐེངས་ 次/ཟླ་བ 月	ཐེངས་ 次/ལོ 年	གཏན་ནས་མེད 从不
		གནས་ཚུལ་དངོས་ལ་གཞིགས་ནས་འབྲི་དགོས་ 按实际情况填写				填 99
5.01 སྲན་ཞོ 豆腐						
5.02 སྲན་ཞོ་སྐམ་པོ 干豆腐						
5.03 སྲན་ཁུ 豆浆						
5.04 སྲན་མའི་རིགས་གཞན 其他豆制品						
5.05 སྲན་ལྗོན 绿豆						
5.06 སྲན་མ་སེར་པོ 黄豆						
5.07 བ་དམ་སྐམ་པོ 干大豆						

5.08 དེ་མིན་ 其他<br>（**请注明：**________）						

E6 སྐྱུར་ཚལ<br>咸菜	ཆ་སྙོམས་ཟས་ཚད་/ཁི<br>平均摄入量/克	ཆ་སྙོམས་ཟས་ཚད་ 平均摄入量/ཐེངས་ 次				
		ཐེངས་<br>次/ཉིན<br>天	ཐེངས་<br>次<br>/གཟའ་<br>འཁོར 周	ཐེངས་<br>次/ཟླ་བ<br>月	ཐེངས་<br>次/ལོ<br>年	གཏན་<br>ནས་མེད<br>从不
		གནས་ཚུལ་དངོས་ལ་གཞིགས་ནས་འབྲི་དགོས་ 按实际情况填写				填 99
6.01 ལ་ཕུག་སྐྱུར་མོ<br>咸萝卜						
6.02 ཡང་ཀའ་སྐྱུར་མོ<br>咸黄瓜						
6.03 ཀྲུ་ཚལ 榨菜						
6.04 བསྔལ་ཚལ 泡菜						
6.05 སྒོག་པ་སྔལ་མ<br>腌蒜						

E7 སིལ་ཟས་དང་ཤུན་<br>མཁྲེགས་ཤིང་ཏོག<br>小吃和坚果	ཆ་སྙོམས་ཟས་ཚད་/ཁི<br>平均摄入量/克	ཆ་སྙོམས་ཟས་ཚད 平均摄入量/ཐེངས་ 次				
		ཐེངས་<br>次/ཉིན<br>天	ཐེངས་<br>次<br>/གཟའ་<br>འཁོར 周	ཐེངས་<br>次/ཟླ་བ<br>月	ཐེངས་<br>次/ལོ<br>年	གཏན་<br>ནས་མེད<br>从不
		གནས་ཚུལ་དངོས་ལ་གཞིགས་ནས་འབྲི་དགོས་<br>按实际情况填写				填 99
7.01སྒོ་ངའི་བག་ལེབ<br>蛋糕						
7.02བག་ལེབ 面包						
7.03 ཏན་ཞིམ 饼干						
7.04 སྐབས་བདེ་ཐུག་<br>པ 方便面						
7.05 ཞོག་བཀྲག<br>薯片						
7.06 དེ་མིན 其他<br>（**请注明：**________）						
7.07 བ་དམ 花生						
7.08 སྟར་ཀ 核桃						
7.09 ལི་ཙེ 栗子						

7. 10 ཁམ་ཚིག 杏仁						
7. 11 ཀྲིན་འབྲ 榛子						
7. 12 ཐང་འབྲ 松子						
7. 13 སྙིང་རྟོག 开心果						
7. 14 ཡའོ་རྟོག 腰果						
7. 15 དེ་མིན 其他<br>（**请注明：**________）						

E8 ཧམ་བུ་རྒྱབ་པའི་ཟས་དང་ཤ་མོའི་ཟས<br>真菌和蘑菇	ཆ་སྙོམས་ཟས་ཚད/ཁེ<br>平均摄入量/克	ཆ་སྙོམས་ཟས་ཚད 平均摄入量/ཐེངས་ 次				
		ཐེངས་<br>次/ཉིན<br>天	ཐེངས་<br>次<br>/གཟའ་<br>འཁོར 周	ཐེངས་<br>次/ཟླ་བ<br>月	ཐེངས་<br>次/ལོ<br>年	གཏན་<br>ནས་མེད<br>从不
		གནས་ཚུལ་དངོས་ལ་གཞིགས་ནས་འབྲི་དགོས་<br>按实际情况填写				填 99
8. 01 ཤ་མོ་སྐམ་པོ<br>干蘑菇						
8. 02 ཤ་མོ་གསོས་པ<br>鲜蘑菇						
8. 03 ཧའེ་ཏེ 海带						
8. 04 སུ་མན་གྱི་ཚལ<br>紫菜						

E9<br>ཚལ་རིགས་གསོས་པ<br>新鲜蔬菜	ཆ་སྙོམས་ཟས་ཚད<br>平均摄入量/ཁེ 克	ཆ་སྙོམས་ཟས་ཚད་ 平均摄入量/次				
		ཐེངས་<br>次/ཉིན<br>天	ཐེངས་<br>次<br>/གཟའ་<br>འཁོར 周	ཐེངས་<br>次/ཟླ་བ<br>月	ཐེངས 次<br>/ལོ 年	གཏན་<br>ནས་མེད<br>从不
		གནས་ཚུལ་དངོས་ལ་གཞིགས་ནས་འབྲི་དགོས་ 按实际情况填写				填 99
9. 01 སྔོ་ཚལ་ཚང་མ<br>全部蔬菜						

བཟའ་བྱའི་སྔོ་ཚལ་གྱི་མིང་དང་སྤྱིའི་ཟས་ཚད་ཀྱི་བརྒྱ་ཆའི་བསྡུར་ཚད
食用蔬菜名称和总摄入量的百分比

ཨང་གྲངས་ 号码	སྔོ་ཚལ་མིང་ 蔬菜名字	ཟས་ཚད་ 频率<br>（ཐེངས་ 次/ལོ<br>年）	སྤྱིའི་ཟས་ཚད་ཀྱི་བརྒྱ་ཆའི་བསྡུར་ཚད<br>占总摄入量的百分比<br>（%）
9. 02	པད་ཚལ་ཆུང་བ 小白菜		

9.03	ལ་ཕུག་དམར་པོ 胡萝卜		
9.04	ཞོག་ཁོག 土豆		
9.05	པད་ཚལ་ཆེ་བ 大白菜		
9.06	སོ་ལོ 青椒		
9.07	སྲན་ལོན 豆芽		
9.08	ཨོ་སྲུན 莴笋		
9.09	མེ་ཏོག་པད་ཚལ 花菜		
9.10	སོ་ལོ་དམར་པོ 甜椒		
9.11	ཏུང་ཀའ 冬瓜		
9.12	ནན་ཀའ 南瓜		

E10 ཤིང་འབྲས 水果	ཆ་སྙོམས་ཟས་ཚད 平均摄入量/ཁེ 克	ཆ་སྙོམས་ཟས་ཚད 平均摄入量/ཐེངས་ 次				
		ཐེངས་ 次/ཉིན 天	ཐེངས་ 次 /གཟའ་འཁོར 周	ཐེངས་ 次/ཟླ་བ 月	ཐེངས་ 次/ལོ 年	གཏན་ནས་མེད 从不
		གནས་ཚུལ་དངོས་ལ་གཞིགས་ནས་འབྲི་དགོས་ 按实际情况填写				填 99
10.01 ཞི་ཀྭ 西瓜						
10.02 རྒུན་འབྲུམ 葡萄						
10.03 ཀུ་ཤུ 苹果						
10.04 ངང་ལག 香蕉						
10.05 ལི 梨						
10.06 ཚ་ལུ་མ 橘子						
10.07 རྒུན་འབྲུམ་སྐམ་པོ་ 葡萄干						
10.08 ཧའ་མིན་ཀྭ 哈密瓜						
10.09 ཁམ་བུ 桃子						
10.10 འབྲུག་མིག 龙眼						
10.11 མངར་ཀུབ 甜瓜						
10.12 འབྲི་ཏ་ས་འཛིན 草莓						
10.13 ལི་ཀྲི 荔枝						
10.14 ཨ་མྲ 芒果						
10.15 ཤིང་ཏོག་པོ་ལུའོ 菠萝						

10. 16 ལྕགས་ཀྱིན 罐头						
10. 17 ཁམ་བུ་སྤྲེ་ཧོའུ 猕猴桃						
10. 18 སྲུ་ཙ་མེ་ཏོག 山楂						
10. 19 ཨ་སྲ 柿子						
10. 20 ཁམ་ཆོག 杏子						
10. 21 རྒྱ་དམ 梅子						
10. 22 ཆི་བི་ཁ 枣子						
10. 23 ཚ་ལུ་མ 橙子						
10. 24 དེ་མིན 其他（请注明：________）						

E11 ཇ་དང་བཏུང་བྱ 茶和饮料	ཆ་སྙོམས་བཏུང་ཚད 平均摄入量/ཁ 克	ཆ་སྙོམས་བཏུང་ཚད 平均摄入量/ཐེངས་ 次				
		ཐེངས་ 次/ཉིན 天	ཐེངས་ 次 /གཟའ་འཁོར 周	ཐེངས་ 次/ཟླ་བ 月	ཐེངས་ 次/ལོ 年	གཏན་ནས་མེད 从不
		གནས་ཚུལ་དངོས་ལ་གཞིགས་ནས་འབྲི་དགོས་ 按实际情况填写				填 99
11. 01ཇ་བསྲུབ་མ 酥油茶						
11. 02ཇ་མངར་མོ 甜茶						
11. 03 ཇ་དངས 清茶						
11. 04 ཁུ་ནག 可乐						
11. 05ཤིང་ཏོག་ཁུ་བ 果汁						
11. 06 འཆོག་ཇ 咖啡						
11. 07ཇ་དམར་པོ 红茶						
11. 08 བ་སྤུའི་མེ་ཏོག་ཇ 茉莉花茶						
11. 09 འོ་ཇ 奶茶						
11. 10 དེ་མིན 其他（请注明：________）						

E12ཀ་ར་དང་བསིང་ཕྱེ 糖和淀粉 མངར་ཆ་དང་གྲོ་ཞིབ	ཆ་སྙོམས་ཟས་ཚད 平均摄入量/ཁི 克	ཆ་སྙོམས་ཟས་ཚད 平均摄入量/ཐེངས་ 次				
		ཐེངས་ 次/ཉིན 天	ཐེངས་ 次 /གཟའ་ འཁོར 周	ཐེངས་ 次/ཟླ་བ 月	ཐེངས་ 次/ལོ 年	གཏན་ ནས་མེད 从不
		གནས་ཚུལ་དངོས་ལ་གཞིགས་ན་སའབྲི་དགོས་ 按实际情况填写				填 99
12.01 ཀ་ར 糖						
12.02 སྦྲང་རྩི 蜂蜜						
12.03མངར་རིགས་ གཞན 其他糖 （**请注明：**______）						
12.04 གྲོ་ཕྱེ 淀粉						

12.05 ཁྱིམ་ཚང་རེ་རེས་ཟླ་རེར་གཤམ་གྱི་དྲི་བ་འདི་དག་འདྲི་དགོས་
每个家庭每个月都要问以下问题，
མི་ག་ཚོད་ཀྱིས་ཁྱིམ་ནང་མཉམ་དུ་ཞལ་ལག་མཆོད་ཀྱི་ཡོད་དམ།
有______ 人在家庭中一起吃饭？
注：མི་རེའི་ཟས་ཚད 个人摄入量(ཁིg/ཉིན 日)=ཁྱིམ་ཚང་ཟས་ཚད 家庭摄入量（རྒྱ་མ 斤/ཟླ་བ 月）×500÷ནང་མི 家庭成员÷30

E13 བཟའ་སྣུམ 食用油	ཟས་ཚད 摄入量（རྒྱ་མ 斤/ཟླ་བ 月/ཁྱིམ 家）	ཟས་ཚད 摄入量/མི 人/ཉིན 日*（ཁི 克/ཉིན 日）
13.01 བ་དམ་སྣུམ 花生油		
13.02 སྲན་སྣུམ 大豆油		
13.03མཉམ་བསྲེས་སྐྱེ་དངོས་སྣུམ 混合植物油		
13.04རྩི་ཤིང་སྣུམ་གཞན 其他植物油 （**请注明：**______）		
13.05སྲོག་ཆགས་སྣུམ་གཞན 其他动物油 （**请注明：**______）		

E14 སྨན་སྣ 香料	ཟས་ཚད 摄入量（རྒྱ་མ 斤/ཟླ་བ 月/ཁྱིམ 家）	ཟས་ཚད 摄入量/མི 人/ཉིན 日*（ཁི 克/ཉིན 日）
14.01ཚྭ 盐		

14.02ཚང་ཡིའུ 酱油		
14.03ཚུའུ 醋		
14.04སྦོ་རྫས 味精		
14.05 དེ་མིན 其他<br>（请注明：________）		

注：མི་སྒེར་ཟས་ཚོད་
个人摄入量(ཁg/ཉིན 日)=
ཁྱིམ་ཚང་ཟས་ཚོད
家庭摄入量（རྒྱ་མ 斤/ཟླ་བ 月）×500÷ནང་མི 家庭成员÷30

<table>
<tr><td rowspan="3">E15རྒྱུན་ཟས་གཞན
其他经常食用的食物</td><td rowspan="3">ཆ་སྙོམས་ཟས་ཚོད
平均摄入量/ཁ
克</td><td colspan="5">ཆ་སྙོམས་ཟས་ཚོད 平均摄入量/ཐེངས་ 次</td></tr>
<tr><td>ཐེངས་ 次
/ཉིན 天</td><td>ཐེངས 次
/གཟའ་
周</td><td>ཐེངས་ 次
/ཟླ་བ 月</td><td>ཐེངས་ 次
/ལོ 年</td><td>གཏན་ནས་
མེད 从
不</td></tr>
<tr><td colspan="4">གནས་ཚུལ་དངོས་ལ་གཞིགས་ནས་འབྲི་དགོས་
按实际情况填写</td><td>填 99</td></tr>
<tr><td>15.01</td><td></td><td></td><td></td><td></td><td></td><td></td></tr>
<tr><td>15.02</td><td></td><td></td><td></td><td></td><td></td><td></td></tr>
<tr><td>15.03</td><td></td><td></td><td></td><td></td><td></td><td></td></tr>
<tr><td>15.04</td><td></td><td></td><td></td><td></td><td></td><td></td></tr>
<tr><td>15.05</td><td></td><td></td><td></td><td></td><td></td><td></td></tr>
</table>

བརྟག་དཔྱད་དུས་ཚོད་ 调查时间：__________ལོ 年________ཟླ་ 月________ཉིན 日
བརྟག་དཔྱད་མཁན་ 调查员：__________ཞིབ་གཉེར་མཁན་ 审核员：__________
